# The role of activating transcription factor 6 in hydroxycamptothecin-induced fibroblast autophagy and apoptosis

**DOI:** 10.1186/s13018-020-02056-z

**Published:** 2021-01-04

**Authors:** Jin Tao, Hui Chen, Xiaolei Li, Jingcheng Wang

**Affiliations:** Department of Orthopedics, Orthopedic Institute, Clinical Medical College of Yangzhou University, Northern Jiangsu People’s Hospital, Yangzhou, People’s Republic of China

**Keywords:** Hydroxycamptothecin, Autophagy, Apoptosis, Activating transcription factor 6

## Abstract

**Background:**

The over-proliferation of fibroblasts is considered to be the main cause of scar adhesion after joint surgery. Hydroxycamptothecin (HCPT), though as a potent antineoplastic drug, shows preventive effects on scar adhesion. This study aimed to investigate the role of activating transcription factor 6 (ATF-6) in the HCPT-induced inhibition of fibroblast viability.

**Methods:**

The cell counting kit-8 (CCK-8) assay, western blot analysis, lentivirus-mediated gene silencing, transmission electron microscopy (TEM) analysis, immunofluorescent staining for autophagy-related protein light chain 3 (LC3) were used to explore the effect of HCPT on triggering fibroblast apoptosis and inhibiting fibroblast proliferation, and the involvement of possible signaling pathways.

**Results:**

It was found that HCPT exacerbated fibroblast apoptosis and repressed its proliferation. Subsequently, endoplasmic reticulum stress (ERS)-related proteins were determined by western blot prior to ATF6 p50 was screened out and reexamined after it was silenced. As a result, ATF6-mediated ERS played a role in HCPT-induced fibroblast apoptosis. Autophagy-related proteins and autophagosomes were detected after the HCPT administration using western blot and TEM analyses, respectively. Autophagy was activated after the HCPT treatment. With the co-treatment of autophagy inhibitor 3-methyladenine (3-MA), both the western blot analysis and the CCK-8 assay showed inhibited autophagy, which indicated that the effect of HCPT on fibroblast proliferation was partially reversed. Besides, the LC3 immunofluorescence staining revealed suppressed autophagy after silencing ATF6 p50.

**Conclusion:**

Our results demonstrate that HCPT acts as a facilitator of fibroblast apoptosis and inhibitor of fibroblast proliferation for curbing the postoperative scar adhesion, in which the ATF6-mediated ERS pathway and autophagy are involved.

## Introduction

Intra-articular scar adhesion commonly occurs following knee arthroplasty, cruciate ligament reconstruction, internal fixation for tibial plateau fractures, and other operations, seriously affecting the postoperative efficacy and daily work of patients due to the limited flexion and extension and the stiffness of the joint [1, 2]. Therefore, effectively preventing postoperative scar adhesion is the priority to improve the success rate of the surgery.

Although the exact mechanism of intra-articular adhesion still remains unclear, it is speculated that the excessive proliferation of fibroblasts in the surgical area is the main cause. Therefore, prohibiting fibroblast proliferation and inducing its apoptosis can be a feasible method against scar adhesion following joint surgery. A previous study has confirmed the preventive effect of HCPT on scar adhesion after knee surgery in rabbits [3]. Furthermore, in our study, HCPT has facilitated fibroblast apoptosis via the activation of ERS and thwarted joint adhesion through PERK and IRE1 signalings, and the role of ATF6 has been highlighted in our experiments.

HCPT as a potent anticancer agent extracted from *Camptotheca acuminata*, a deciduous plant of Davidiaceae, effectively inhibits DNA topoisomerase I [4], an important target of cancer chemotherapy. As a result, it has been widely used for a variety of cancers, such as hepatocellular carcinoma, gastric cancer, and digestive tract tumors [5, 6].

The balance between intracellular protein biosynthesis and catabolism is a physiological process of cell survival; otherwise, cell death may occur. At present, necrosis, apoptosis, and autophagic cell death are listed as three major types of cell death, of which apoptosis, that is, type I programmed cell death, is characterized by apoptotic bodies [7] and autophagic cell death, type II programmed cell death, by autophagy [8].

ERS, resulting from incorrect protein folding and the aggregation of unfolded proteins in endoplasmic reticulum lumen and a disturbance of calcium balance, can activate signaling pathways such as unfolded protein response (UPR) and caspase-12-mediated apoptotic pathway [9]. Activated transcription factor 6 (ATF-6) is an ER sensor protein of endoplasmic reticulum (ER) and an important regulator of apoptotic and autophagy pathway triggered by ERS.

Autophagy is a highly conserved metabolic pathway that scavenges, degrades, and absorbs macromolecules and damaged organelles in mammals, consisting of macrophage, microautophagy, and chaperone-mediated autophagy. The timely removal of incorrectly synthesized or folded proteins in cells except for damaged or senescent organelles is another major biofunction of autophagy for maintaining the self-stabilization of cells [10].

ATF6 has been corroborated to act as a regulator of autophagy—for instance, avian metapneumovirus subgroup C (aMPV-C) induces autophagy through the ATF6 UPR pathway [11]. However, whether the ATF6 pathway involves in the preventive effect of HCPT on joint adhesion by regulating autophagy is not clear. The purpose of this study was to investigate the potential mechanism of HCPT in preventing scar adhesion after joint surgery.

## Materials and methods

Primary human fibroblast cell line was purchased from GuangZhou Jenino Biotech Co., Ltd. We cultured fibroblasts in the Dulbecco’s Modified Eagle’s medium (DMEM; Gibco, USA) containing 20% fetal bovine serum (FBS; Gibco), 100 U/ml penicillin, and 100 U/ml streptomycin (Thermo, USA). The cells were incubated in an incubator at 37 °C and 5% CO_2_. The fibroblasts steadily growing to the 4th and 7th generations were used for cell experiments. This experimental study was approved by the Ethics Committee and Research Committee of Northern Jiangsu People’s Hospital.

### HCPT treatment

HCPT was purchased from Dalian Meilun Biological Technology Co., Ltd. Fibroblasts were cultured in 6-well plates, 96-well plates, or 10-cm-diameter culture dishes. When the cells were cultured to about 80%, they were rinsed with phosphate buffer saline (PBS) for three times. The selection of HCPT concentrations was based on our previous articles and experiments. Human fibroblasts were administered with HCPT and the controls were incubated with PBS. Experimental and control cells were then collected for subsequent cell experiments.

### Cell viability assay

The CCK-8 assay (Dojindo, Tokyo, Japan) was performed for fibroblast viability. Fibroblasts in the 5th to 7th generation were selected and seeded in two 96-well plates. In one group, when the cells were grown to about 80%, they were treated with different concentrations of HCPT, and the metabolites were first removed by washing with PBS buffer. After that, 100 μL DMEM and 10 μL CCK-8 reagent were added before a 2-h incubation. The optical density was read at 450 nm using a microplate bance reader (Bio-Tek, Elx 800, USA). The cell viability was calculated on the basis of the reference manual.

### Western blot analysis

The fibroblasts administered by HCPT at different concentrations or at a single concentration at various time points were collected before target proteins were quantitated by western blot analysis. Firstly, the collected fibroblasts were treated on ice and lysed in radioimmunoprecipitation assay (RIPA) buffer (Beyotime, Hangzhou, China). Secondly, cells were sonicated and centrifuged for collecting the total protein. Thirdly, the concentration of the total protein was determined using a BCA protein assay kit (Beyotime, Hangzhou, China). The total protein (40 μg) from lysates were loaded in each lane, separated by a 5–12% sodium dodecyl sulfate-polyacrylamide gel electrophoresis (SDS-PAGE) and then transferred to polyvinylidene difluoride (PVDF) membranes (Millipore, Bedford, MA) for 1.5 h at 200 mA. PDVF membranes were blocked for 2 h at room temperature with 5% skimmed milk and were washed in tris-buffered saline and Tween 20 (TBST) solution. The membranes were incubated with primary and secondary antibodies continuously in accordance with the reference manual. Primary antibodies encompassed anti-Cyclin D1, anti-PCNA, anti-Bax, anti-Bcl2, anti-78 kDa glucose-regulated protein (GRP78), anti-CHOP, anti-ATF6 p50, anti-p62, anti-Beclin-1, anti-LC3-II/LC3-I, and anti-*β*-actin antibodies (diluted 1:1000), and secondary antibodies incorporated anti-mouse or anti-rabbit IgG (diluted 1:5000). All this was purchased from Cell Signaling Technology (Beverly, MA, USA).

### Lentivirus-mediated gene silencing

ATF6 silencing plasmid was purchased from Shanghai Genechem Co., Ltd. (Genechem, China). During vector production, 293 T cells were seeded in 6-well plates at a ratio of 80 to 90%, and subsequently, plasmids were introduced into 239 T cells. The supernatant containing lentivirus was collected and mixed with 10 ng/mL polybrene (Gibco, USA) for application in fibroblasts. Afterward, infected fibroblasts were screened with 1 μg/mL puromycin (Sigma, USA).

### TEM analysis

The process of autophages was observed using TEM. Cells were collected after HCPT treatment, fixed in 2.5% glutaraldehyde stationary solution at 4 °C overnight, and then fixed in 1% OSO4 solution for 1 h. They were dehydrated gradiently with ethanol and embedded in Epon 812. The cells stained with uranyl acetate and lead citrate were finally observed and calculated using the Philips Tecnal 10 TEM.

### Immunofluorescent staining for LC3

After HCPT treatment at different doses, the cells were fixed with 4% paraformaldehyde for 10 min at room temperature, washed and permeated with 0.1% Triton X-100 for 15 min, and then were incubated with anti-LC3 antibody (diluted at 1:100) overnight, washed and incubated with anti-rabbit IgG H&L (Alexa Fluor 555) antibody (1:200 dilution) (Abcam, British) for 1 h at room temperature. The cells were stained with DAPI, washed with PBS, and observed and photographed under a fluorescence microscope.

### Statistical analysis

The data were analyzed using the SPSS 19.0 statistical software. All data were expressed as mean ± standard deviation (SD). The one-way ANOVA was used to compare the results of each group. Statistical significance was defined as a *P* value of < 0.05.

## Results

### The effect of HCPT on fibroblasts

**Fig. 1:**
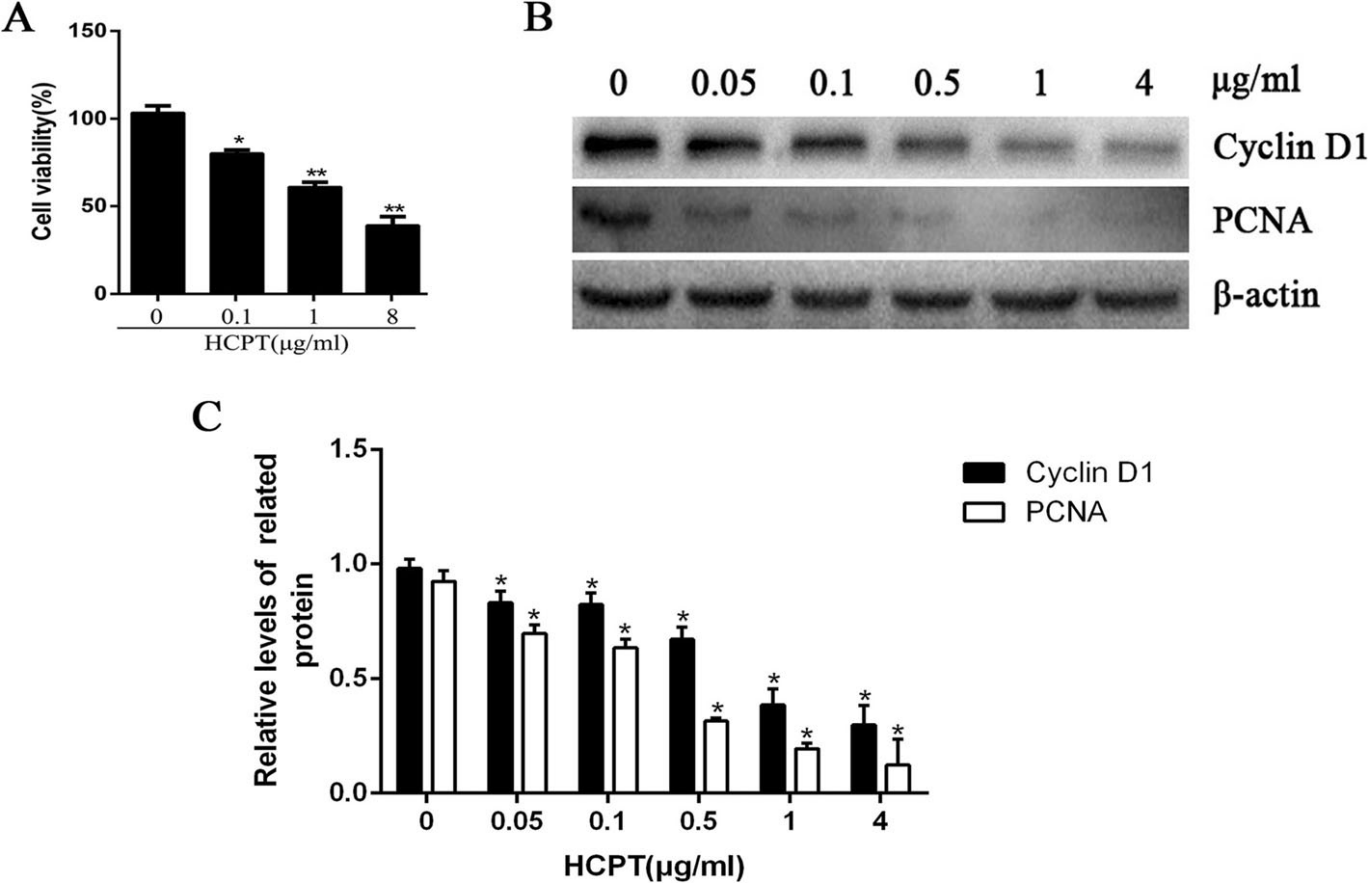
HCPT inhibits fibroblast proliferation. **a** Dose-dependent effects on fibroblasts after treated with different concentrations of HCPT, the viability was determined by CCK-8 assay, *P < 0.05, **P < 0.01 vs 0 μ/ml group. **b**-**c** The expression of Cyclin D1and PCNA was determined by western blot analysis in human fibroblast cells treated with 0, 0.05, 0.5, 0.1, and 4 μg/mL HCPT, *P < 0.05 vs 0 μ/ml group. β-actin was used as a control. These experiments were performed in triplicate

Our previous studies have shown that HCPT triggered fibroblast apoptosis in a dose- and time-dependent manner. Results of the CCK-8 assay (Fig. 1a) indicated that the viability of fibroblasts significantly decreased as the concentration of HCPT gradually increased, showing a prominent concentration-dependent trend. The western blot analysis (Fig. 1b and c) showed that expressions of proliferation-related proteins PCNA and Cyclin D1 were downregulated as the concentration of HCPT rose, in a dose-dependent manner. These results suggested that HCPT inhibited the viability of fibroblasts possibly by repressing its proliferation and triggering apoptosis.

### HCPT activated ERS-mediated apoptosis through the ATF6 pathway

**Fig. 2:**
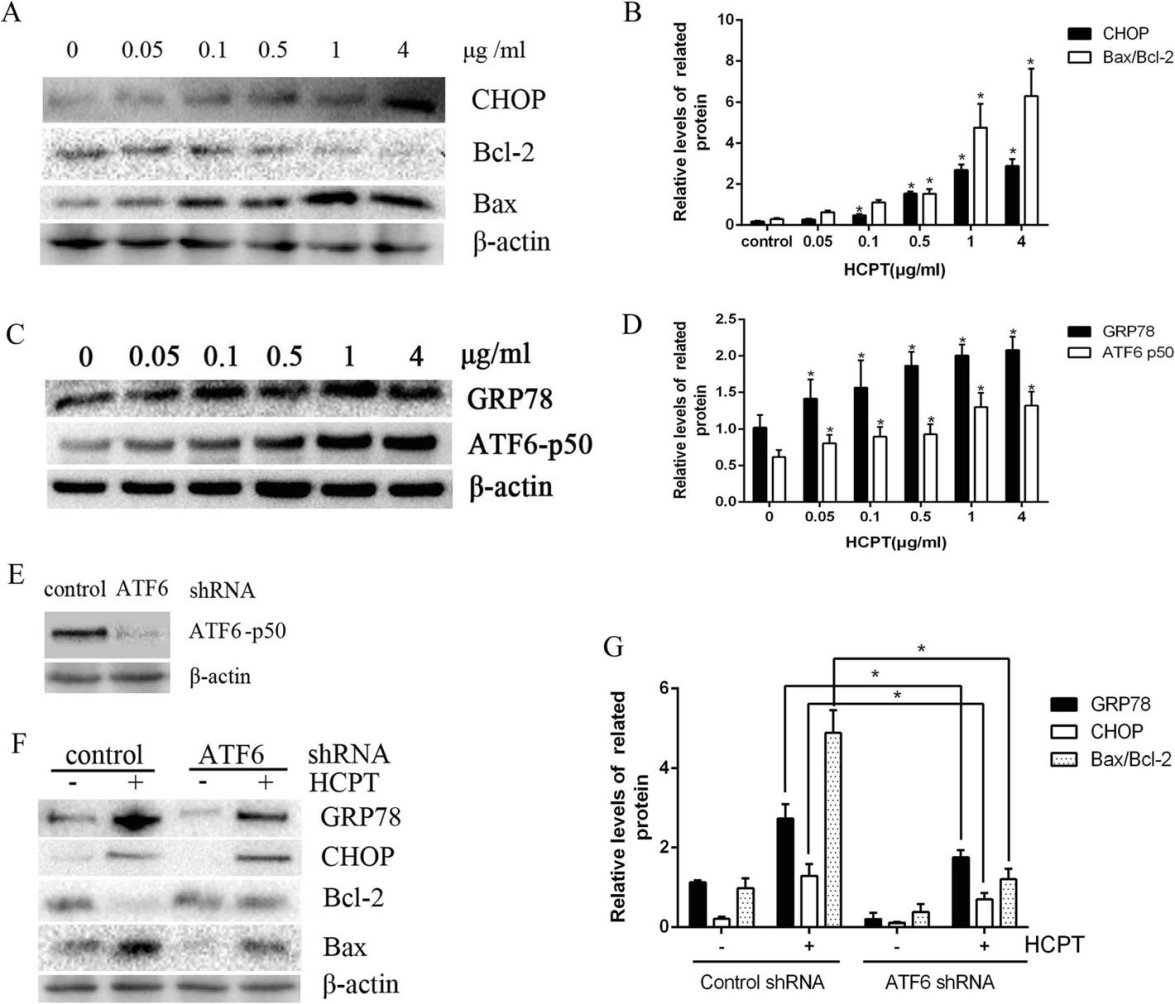
HCPT induces apoptosis of fibroblasts through ATF6-mediated ERS. **a**-**b** The expression of CHOP, Bcl-2, and Bax was determined by western blot analysis in human fibroblast cells treated with 0, 0.05, 0.5, 0.1, and 4 μg/mL HCPT. *P < 0.05 vs 0 μ/ml group. β-actin was used as a control. **c**-**d** The expression of GRP-78 and ATF6 p50 were determined by western blot analysis, *P < 0.05 vs 0 μ/ml group. **e** Silenced ATF-6 p50 expression was verified by western blot analysis. **f**-**g** The expression levels of the ER stress markers GRP78 and CHOP and the downstream Bcl-2 and Bax were determined by western blot in ATF-6-knockdown and control group after treatment with or without 1 μg/mL HCPT, *P < 0.05. These experiments were performed in triplicate

Expressions of ERS- and apoptosis-related proteins after administrations with different doses of HCPT were detected (Fig. 2a and b). The western blot analysis revealed that Bax and CHOP levels were elevated, while the level of Bcl-2 declined gradually, indicating that cell apoptosis by HCPT was underway. The expression of ERS-associated protein GRP78 was upregulated with an increasing concentration of HCPT—indicating the enhanced ER pathway, synchronized with an escalating level of ATF6 p50 (Fig. 2c-d). This suggested that ATF-6 could play a role through the ER pathway. We then built stable ATF-6-silenced fibroblasts. The western blot analysis showed that ATF-6 p50 was successfully knocked down (Fig. 2e). With the administration of HCPT, the ATF-6 knockdown group presented lower levels of GRP78, CHOP, and Bax but a higher level of Bcl-2 than the control group (Fig. 2f and g). These results indicated that HCPT triggered apoptosis of human fibroblasts through ATF6-mediated ERS.

### HCPT suppressed fibroblast proliferation by inducing autophagy

**Fig. 3:**
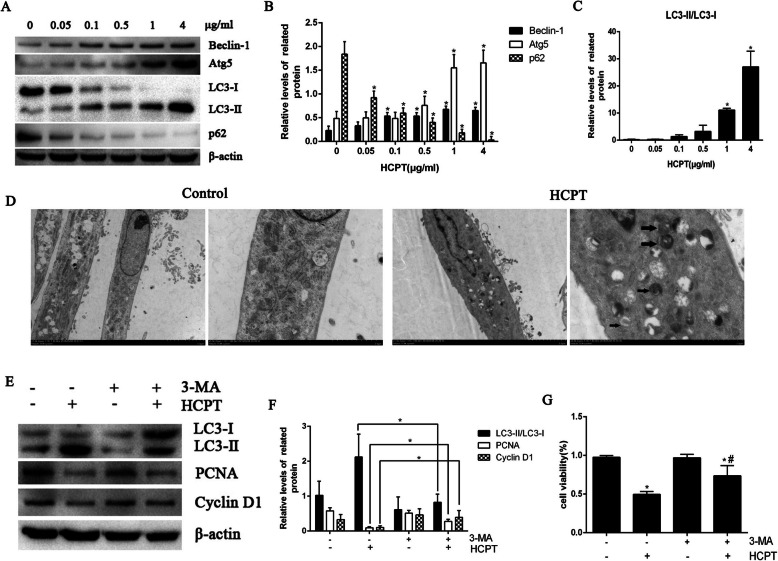
HCPT inhibits fibroblast proliferation by inducing autophagy. **a**-**c** The expression of Atg5, Beclin-1, LC3, p62 was determined by western blot analysis. β-actin was used as a control, *P < 0.05 versus 0 μg/mL. **d** TEM results showed that autophagic bodies appeared after HCPT treatment. Black arrows indicate autophagic bodies. **e**-**f** After treated with or without 3-MA, the fibroblasts were treated with 1 μg/mL HCPT, then detected by western blot analysis with antibodies specific for LC3, PCNA, and Cyclin D1, *P < 0.05. **g** After treated with or without 3-MA, the fibroblasts were treated with HCPT as described before and then cell viability was measured after 24 h. *P < 0.05 versus control group (0 μg/mL), #*P* < 0.05 versus HCPT treated only group (1 μg/mL). These experiments were performed in triplicate

The western blot analysis showed that autophagy-related proteins Atg5 and Beclin-1, and the LC3-II/LC3-I ratio increased while p62 decreased after HCPT administrations at different concentrations, suggesting that autophagy was activated (Fig. 3a-c). The morphology of fibroblasts treated with HCPT was observed using the TEM analysis—the gold standard for detecting autophagy, and autophagosomes were found (Fig. 3d). To verify the involvement of autophagy in the HCPT-induced inhibition of fibroblast viability, fibroblasts were co-treated with the autophagy inhibitor 3-MA and autophagy was found to be inhibited, which meant the inhibition of HCPT on fibroblasts proliferation was curbed (Fig. 3e-f). The CCK-8 assay showed the similar results (Fig. 3g). All this suggested that HCPT inhibited fibroblast proliferation by triggering autophagy.

### Roles of ATF6 in autophagy and apoptosis

**Fig. 4:**
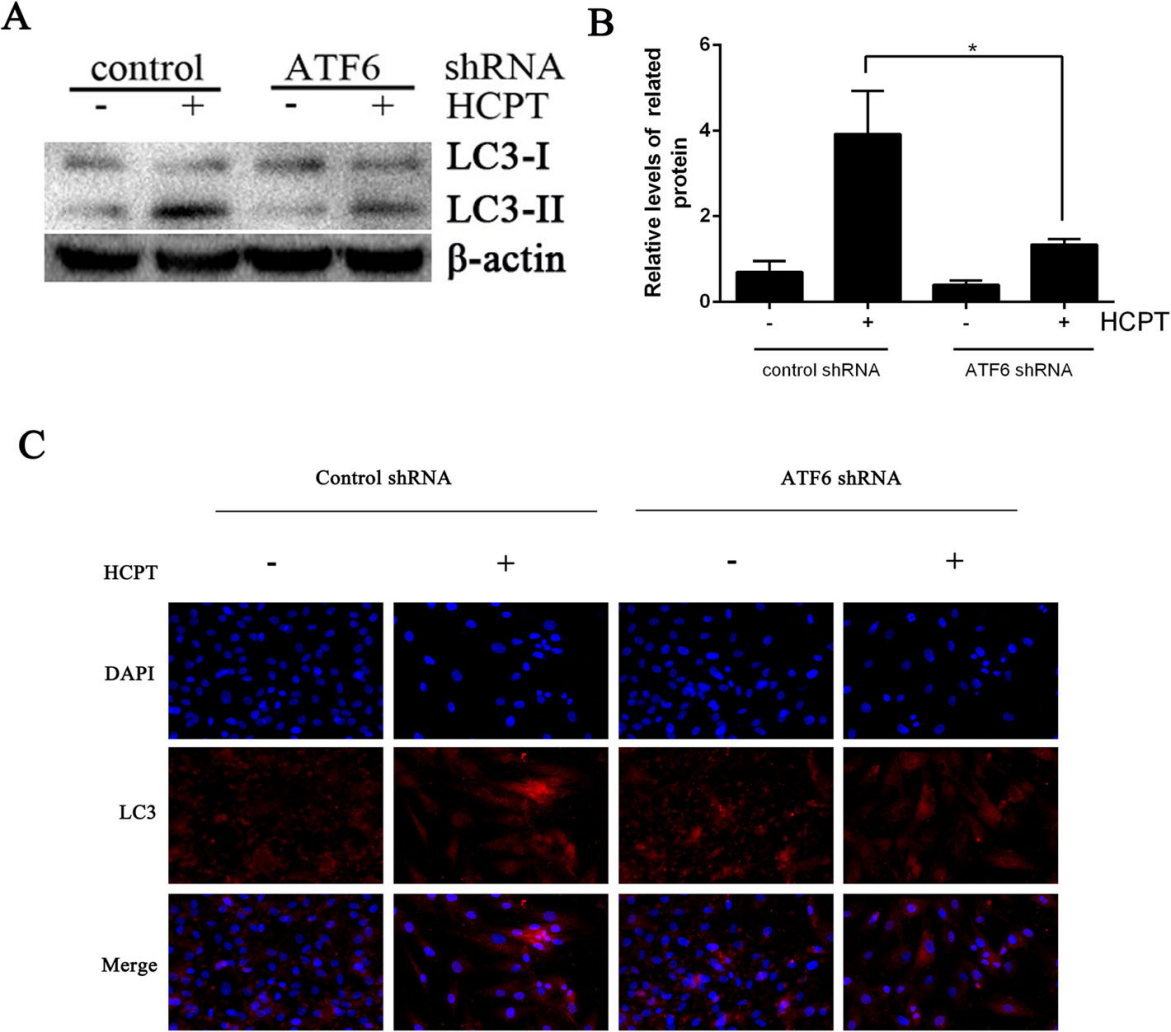
HCPT regulates autophagy through ATF6 pathway. **a**-**b** The expression levels of the autophagy markers LC3 were determined by western blot in ATF-6-knockdown and control group after treatment with or without 1 μg/mL HCPT, *P < 0.05. **c** ATF-6-knockdown and control group after treatment with or without 1 μg/mL HCPT were measured by LC3 immunofluorescence, the accumulation of LC3 punctate dots was decreased after silencing ATF6 gene. These experiments were performed in triplicate

As shown in Fig. 2f-h, after silencing ATF6, the apoptotic effect of HCPT on fibroblasts was weakened. And the analysis for autophagy-related proteins revealed that the LC3-II/LC3-I ratio decreased (Fig. 4a and b). The result was reconfirmed in the LC3 immunofluorescence (Fig. 4c). These results suggested that despite the role of the ATF6 pathway in ERS-induced apoptosis, it is also involved in the inhibition of fibroblast proliferation via the autophagy pathway.

## Discussion

Intra-articular scar adhesion as a common complication of knee surgery often results in the knee-joint dysfunction and limitation of movement, further weakens the therapeutic efficacy of surgical treatments, and affects the quality of life in patients [12]. As the specific pathological mechanism of intra-articular scar adhesion is still unclear, the method by suppressing fibroblast proliferation and accelerating its apoptosis may be feasible to protect against the procedure.

HCPT is a kind of camptothecin analog extracted from *C. acuminata*. Owing to its potent biological activity with less toxicity and side effects on malignant tumors, it has been developed as the first-line agent in the management of multiple tumors [13]. Such a topoisomerase I inhibitor can inhibit DNA synthesis for initiating tumor cell apoptosis [14]. Previous studies have found that HCPT can inhibit fibroblast proliferation and accelerate its apoptotic rate [15], and have confirmed its inhibitory effects on scar adhesion following laminectomy and knee surgery [3, 16]. In this study, we find that HCPT facilitates ERS-induced fibroblast apoptosis through the PERK and IRE1 pathways.

In addition to that, the CCK-8 assay reveals the gradually diminished viability of fibroblasts after the HCPT administration in a dose-dependent manner. To explore the underlying mechanism, proliferation-related proteins have been detected by western blot. And HCPT also triggers fibroblast apoptosis except for its inhibitory effect on fibroblast proliferation.

ER is the largest organelle in eukaryotic cells. When cells are subjected to hypoxia, oxidative stress, and viral infection, its biofunction can be destroyed, leading to the accumulation of erroneous proteins in ER [17]. Previous studies have proven that UPR is the most important signaling pathway in the ERS response pathway mainly mediated by three ERS sensors: PERK, IRE-1, and ATF-6, of which ATF-6 is a type II transmembrane protein on ER [18]. When ERS is triggered and strengthened, ATF-6 (ATF6 p90) dissociates from GRP78/Bip and splits into ATF-6p50 and ATF-6p40, of which the former enters the nucleus to activate downstream proteins like CHOP and induces apoptosis thereby [19].

In this experiment, the initiation and enhancement of UPR reaction by HCPT activates and facilitates ATF-6 to dissociate from GRP78/Bip and split. This explains the downregulated ATF-6 p90, upregulated ATF6-p50 and the increased downstream protein CHOP, and the Bax/Bcl-2 ratio. It indicates that ERS involves in the HCPT-induced apoptosis of fibroblasts possibly through the ATF-6 pathway. To validate the hypothesis, we further knockdown ATF-6 and the number of apoptotic fibroblasts significantly decreases even after the HCPT administration at 1 μg/mL. Meanwhile, both ER-related protein levels and the Bax/Bcl-2 ratio decrease, which confirms that HCPT promotes fibroblast apoptosis via the ATF-6 pathway.

Autophagy is a process of intracellular degradation by transporting damaged, aged, or dysfunctional proteins and organelles into lysosomes, followed by digestion and degradation. In organisms, autophagy is a conservative process. Apoptosis and autophagy are two different forms of programmed cell death, which have some differences in morphology, biochemical indicators, and the process of regulating cell death. However, they are not two completely independent processes. Many studies have shown that their functions also interact and restrict each other in some cases: (1) Autophagy and apoptosis cooperate with each other to promote cell death. (2) Autophagy can inhibit apoptosis by promoting cell survival. (3.) Autophagy, although it does not lead to cell death, is involved in the process of apoptosis. However, the specific mechanism of the interaction between autophagy and apoptosis is not very clear. With the deepening of research, at the molecular level, many genes play an important role in the two pathways of apoptosis and autophagy, such as ATG5, p53, and Bcl-2 [20–22]. Studies support ERS as an inducer of autophagy, and PERK and IRE1 have been proven to regulate ERS-mediated autophagy [23–26]. However, how ATF6 mediates ERS-induced autophagy is still a problem.

Analyses for autophagy-associated proteins find that HCPT also induces autophagy, which is reconfirmed by the TEM analysis. To investigate the role of autophagy in the HCPT-induced inhibition of fibroblast proliferation, the autophagy inhibitor 3-MA has been adopted. The western blot analysis reveals partially reversed suppressive effects of HCPT on fibroblast proliferation after autophagy inhibition, which is supported by the enhanced viability of fibroblasts in the CCK-8 assay. All these suggest that HCPT inhibits fibroblast proliferation by triggering autophagy. ATF6 is a classical pathway of ERS. These results indicate that ATF6-mediated ERS may participate in HCPT-induced fibroblasts autophagy. This effect of ATF6 has been verified in ATF6-silenced cell models using western blot and immunofluorescence light staining for the detection of autophagy-related protein LC3. That is, silencing ATF6 can inhibit the occurrence of autophagy; however, the exact role is not yet clear.

## Conclusion

In conclusion, we find that HCPT represses fibroblast levels in a two-pronged molecular approach: facilitating apoptosis and suppressing proliferation. ATF6 involves in both the occurrence of autophagy and ERS-mediated apoptosis. Nonetheless, our experiment has only explored an underlying mechanism of HCPT affecting fibroblast viability, and the specific relationship between apoptosis and autophagy needs to be further studied.

## Data Availability

The datasets supporting the conclusion of this article are included within the article and its supplementary materials.

## Abbreviations

ATF-6: Activating transcription factor 6
HCPT: Hydroxycamptothecin
CCK-8: Cell counting kit-8
ERS: Endoplasmic reticulum stress
3-MA: 3-Methyladenine
LC3: Light chain 3
UPR: Unfolded protein response
GRP78: 78 kDa glucose-regulated protein 78
CHOP: C/EBP homologous protein
PERK: PRKR-like ER kinase
DMEM: Dulbecco’s Modified Eagle’s medium
FBS: Fetal bovine serum
PBS: Phosphate buffer saline
RIPA: Radioimmune deposition
SDS-PAGE: Sodium dodecyl sulfate-polyacrylamide gel electrophoresis
PVDF: Polyvinylidene difluoride
TBST: Tris-buffered saline and Tween20
TEM: Transmission electron microscopy
